# Multinational patterns of seasonal asymmetry in human movement influence infectious disease dynamics

**DOI:** 10.1038/s41467-017-02064-4

**Published:** 2017-12-12

**Authors:** Amy Wesolowski, Elisabeth zu Erbach-Schoenberg, Andrew J. Tatem, Christopher Lourenço, Cecile Viboud, Vivek Charu, Nathan Eagle, Kenth Engø-Monsen, Taimur Qureshi, Caroline O. Buckee, C. J. E. Metcalf

**Affiliations:** 1 Department of Epidemiology, Johns Hopkins Bloomberg School of Public Health, 615 North Wolfe Street, Baltimore, MD 21205 USA; 2 WorldPop, Department of Geography, University of Southampton, University Road, Southampton, SO17 1BJ UK; 3 Flowminder Foundation, Roslagsgatan 17, SE-11355 Stockholm, Sweden; 4 Clinton Health Access Initiative, 383 Dorchester Avenue Suite 400, Boston, MA 02127 USA; 5 Fogarty International Center, National Institutes of Health, 31 Center Drive, Bethesda, MD 20892 USA; 6 Department of Epidemiology, Harvard T.H. Chan School of Public Health, 677 Huntington Avenue, Boston, MA 02115 USA; 7 Telenor Research, Snarøyveien 30, N-1360 Fornebu, Norway; 8 Center for Communicable Disease Dynamics, Harvard T.H. Chan School of Public Health, 677 Huntington Avenue, Boston, MA 02115 USA; 9 Department of Ecology and Evolutionary Biology, Princeton University, 106A Guyot Lane, Princeton, NJ 08544 USA; 10 Woodrow Wilson School, Princeton University, Robertson Hall, Princeton, NJ 08544 USA

## Abstract

Seasonal variation in human mobility is globally ubiquitous and affects the spatial spread of infectious diseases, but the ability to measure seasonality in human movement has been limited by data availability. Here, we use mobile phone data to quantify seasonal travel and directional asymmetries in Kenya, Namibia, and Pakistan, across a spectrum from rural nomadic populations to highly urbanized communities. We then model how the geographic spread of several acute pathogens with varying life histories could depend on country-wide connectivity fluctuations through the year. In all three countries, major national holidays are associated with shifts in the scope of travel. Within this broader pattern, the relative importance of particular routes also fluctuates over the course of the year, with increased travel from rural to urban communities after national holidays, for example. These changes in travel impact how fast communities are likely to be reached by an introduced pathogen.

## Abstract (web summary)

Fine scale mobile phone data is improving capacity to understand seasonal patterns in human movement. Here, the authors use multi-year movement data across three nations, as well as a model of pathogen spread, to understand the consequences of seasonal travel for disease dynamics.

## Introduction

The connectivity of human populations defines the spread of infectious diseases: cities that are visited frequently experience more introductions of vector borne^1,2^ and directly transmitted^3,4^ infections. Estimates of mobility have been used to inform targeting of control efforts^5^ and to characterize the spatial spread of emergent pathogens^2,6–8^, demonstrating the public health implications of human connectivity. Despite the connection between travel and infectious disease spread, one major feature of human mobility has been neglected in most applications to date: human mobility is generally seasonal^9–12^. This phenomenon has been attributed to the intersection of climatic, economic, and social drivers and occurs in countries across the globe. Environmental changes over the course of the year define the timing of agricultural, livestock, and fishery activities^13–16^; and these drivers can in turn shape travel for subsistence (e.g., to water sources during dry seasons^17,18^) and economic activity (e.g., seasonal, migratory labor^15,19–21^). Social drivers such as religious holidays through to school terms also play a substantial role^22^.

Seasonal travel has wide-ranging health implications. Beyond re-introducing pathogens into locations where they have otherwise been controlled, seasonal mobility and resulting human aggregation may also increase transmission of directly transmitted pathogens^8^. Term-times, when children are in schools, are thought to be associated with higher measles^23^, rubella^24^, and influenza transmission^25,26^; the dry seasons when agricultural workers return to urban settings are also associated with higher measles transmission in Niger^27^. Seasonal movement might also coincide with periods of low nutritional status (e.g., the “hunger season”^28^), and is likely to challenge the ability of existing health systems to reach people, particularly in resource poor settings^29^.

Of course, travel represents one among a diversity of ways by which health outcomes can be affected seasonally^30^. Seasonal climatic conditions can directly affect pathogen transmission, by modulating vector biology via temperature effects on life history^31^ or extension of suitable habitat by flooding during the rainy season^32^; or by altering how viral particles fall out of the air^33^ and thus changing transmission of directly transmitted pathogens. Additionally, control efforts, which represent one of the largest footprints on infectious disease incidence globally^34^ tend to focus on time periods in which transmission is most intense (e.g., provision of bed-nets may be concentrated during the season of greatest mosquito abundance) This diverse set of potentially interacting drivers of seasonal fluctuations in infectious disease is perhaps in part responsible for how rarely the impact of seasonal travel has been quantified, despite its ubiquity and evidence for its applied importance^24,27^. However, the single biggest barrier to date has been data availability.

Until recently, available data, such as travel history surveys, border crossing counts, GPS logger, and commuting data, could only capture a snapshot of travel over a smaller geographic area, subpopulation, or time frame, limiting their utility for understanding broad patterns of seasonal travel, as this requires high-resolution, comparable data^9,35^. Further, in many low-income settings, such data are scarce. Novel, and often digital, data sources have driven a change in our ability to tackle the question of seasonal mobility. For example, flows of airline data indicates that seasonal fluxes in national and international travel are driven by national holidays or tourism^36^; and recently, mobile phone data have been used to extract subscriber mobility patterns at national spatial scales^1,5,24,37^. Using the location and timing of mobile communications, subscriber travel patterns have been inferred and used to understand general mobility patterns^38,39^ as well as the spread of infectious diseases^2,5,24,37,40,41^. These data sets provide a powerful window onto spatio-temporal variation in travel^1^. However, to date, these have primarily been used to characterize overall patterns of movement, and have yet to be used to address the question of seasonal travel in depth.

Here, we compared mobile phone data from three countries—Kenya, Namibia, and Pakistan—to characterize commonalities and differences underlying human patterns of mobility. These data sets have been previously analyzed to quantify broad travel patterns in each country^1,2,5^, however the seasonal dimension has not been systematically and comparatively analyzed, and directional aspects of mobility have not been previously investigated. In Namibia, where data were available for multiple years, we show clear, repeatable seasonal patterns in travel between years. In the remaining two countries, the length of the data set (with only a year or less available) limited our ability to identify repeatable seasonal patterns of mobility. However, commonalities between seasonal trends in these data, and those that emerged from the longer time series available from Namibia suggest consistent drivers of seasonal travel, and we thus use this term in subsequent discussion. We evaluate the role of previously suggested drivers of mobility across a unique combination of geographies in Africa and Asia from extremely rural low-density contexts (Namibia) to one of the most densely populated countries (Pakistan), investigating both the amount and direction of travel and their seasonal fluctuations, identifying clear switches in direction over the course of the year. Building on this, we characterize the possible implications of seasonal travel on the spread of infectious disease using a simulation model of geographic spread, accounting for directional asymmetries, seasonal variation in travel, and pathogen characteristics. We further discuss implications for timing of control and outbreak preparedness.

## Results

### Country-wide mobility patterns

**Fig. 1:**
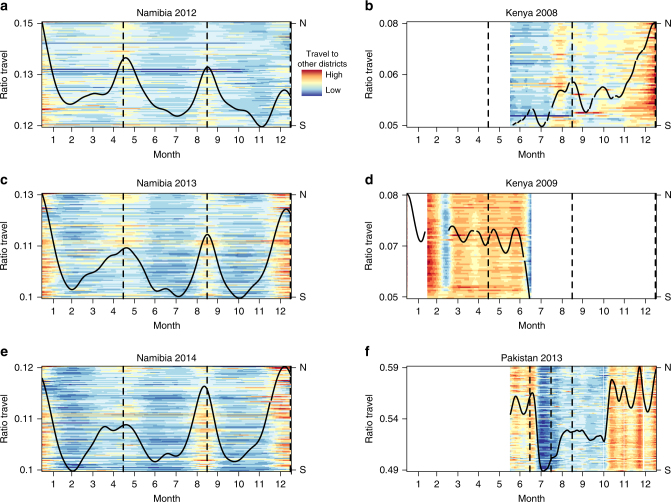
The ratio of trips between districts vs. within a district per day in each country. The ratio of travel to other districts (see Supplementary Fig. 1 for a map of districts) vs. stationary trips per district is shown for each day in each data set by year. Data from each district are shown in each row and ordered according to latitude (North–topmost, South–bottom most). The color represents the percentage of travel ranging from red (high value) to blue (low value). Coloring is scaled to capture the range for each country. Data for Namibia in **a** 2012, **c** 2013, and **e** 2014 are shown. Data for Kenya in **b** 2008 and **d** 2009 are shown. Pakistan data for **f** 2013 are shown. The country median ratio of travel is shown in black (smoothed using a spline), and for each country/year combination echoes underlying color fluctuations over the year, indicating consistent fluctuations in magnitude of travel across spatial scales. School terms are shown as dashed lines

Using previously described methods to extract mobility^1,5^ from mobile phone call data records (CDRs, see Methods, Supplementary Fig. 1), we first characterized country-wide seasonal changes in the magnitude of travel using data from Kenya, Namibia, and Pakistan (Table 1), defined as the proportion of subscribers that traveled on subsequent (e.g., between day i and day i + 1) days. We observe differences in the magnitude of travel between countries: the mean percentage of the population traveling between subsequent days for the entire country was lowest in Kenya (mean: 5%, 95% quantile interval (2–8%)), intermediate in Namibia (13% (11–18%)), and substantially higher in Pakistan (33% (31–36%)), see Supplementary Table 1, Supplementary Figs. 2–3. Beyond broad national differences, all countries showed striking variation over the course of the time series available (Fig. 1). Major national holidays drive particularly strong fluctuations (dashed lines, Fig. 1), with increased travel around Christmas in Kenya and Namibia (December), and decreased travel during Ramadan in Pakistan (July–August); although for Pakistan the lack of a full year of data means that we cannot compare this to the magnitude of travel between January and May. While the largest increase in travel volume happens around Christmas for Kenya and Namibia, the percent of the population traveling peaks roughly three times during the course of the year (highest peak month (December): Kenya ~14% above the average, Namibia ~9% above the average) in line with school term holidays (school breaks shown as a dashed line). The data also suggest a high volume of travel around Christmas in Pakistan, although again, the full year is not available for comparison. The longer Namibian time series allows us to confirm that observed patterns are consistent between years and indeed seasonal.

**Table 1:** An overview of the mobile phone data and geographic characteristics of the three countries analyzed

	**Kenya**	**Namibia**	**Pakistan**
Population	40 M	2.3 M	182 M
Total area	225,000 sq miles	319,000 sq miles	307,000 sq miles
Population/area	178 per sq miles	7.2 per sq miles	592 per sq miles
District population (mean, 1–3rd quantile)	416 K (216–504 K)	20 K (12–25 K)	570 K (202–709 K)
Average district area	11,492 sq miles	7875 sq miles	25,252 sq miles
*Mobile phone data*			
Time frame of data	June 2008–June 2009, excluding February 2009	October 2010–April 2014	June 2013–December 2013
Total number of subscribers in the data	14,816,521	See Supplementary Information	39,785,786
Number of districts (admin 2 level) with mobile phone towers	69	105	113
Percentage of districts with a mobile phone tower	100%	87%	88%

Across all countries, we analyze possible correlates of seasonal travel including social determinants (school terms and national holidays), climatic variables (rainfall and temperature, Supplementary Fig. 4), and an effect of location (e.g., district of origin) using lasso regression to constrain model complexity. We fitted lasso models using cross-validation via the glmnet package in R to minimize the mean-squared error (Supplementary Note 1; Supplementary Table 2). Most variables were retained in all three countries in the model identified under global cross-validation, but social variables (holidays in all three countries and school terms in Namibia) had by far the largest effects with coefficients at least double of the next coefficients. In Pakistan, all variables had small coefficients suggesting that these factors were not able to predict peaks in travel (leading to a small regularization coefficient). This may partly reflect the limited timeframe of these data. In Kenya and Namibia, the coefficient on temperature was negative (although small in Namibia), suggesting that climatic variables may play a smaller, but consistent role in these locations.

**Table 2:** The results from the lasso regression

**Variable**	**Kenya**	**Namibia**	**Pakistan**
(Intercept)	−2.98	−2.33	0.438
School terms		0.0476	
Temperature	−0.132	−0.0014	−0.0004
Precipitation	0.00088	0.00013	
Location	−0.001	0.0034	0.0004
Holiday	0.218	0.058	−0.005

In each country, we performed a lasso regression to predict the monthly average ratio of travel per district using social (school terms), climatic (temperature and precipitation), and a location variable. In all three countries, the social variable (holiday or school terms) was the largest factor

**Fig. 2:**
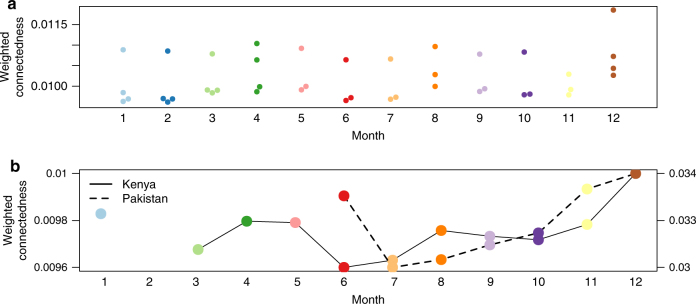
The connectivity per month. For each month, connectivity in each country was measured using the weighted connectedness that takes into account the total number of trips between districts relative to density of edges (Methods). The monthly connectivity values in **a** Namibia, shown as a beeswarm plot with a point for each year, and **b** Kenya (solid line, left axis) and Pakistan (dashed line, right axis) shown for the duration of the time series. All three countries were the most connected in December

To explore how these seasonal fluctuations in mobility translate into seasonal fluctuations in country-scale connectivity, we used data on the total number of trips in each month to construct connectivity networks. Kenya and Namibia were the most connected in December and least connected in June (Fig. 2), although this varied slightly by year in Namibia. Interestingly, in line with the seasonal pattern of fluctuations, we find that Pakistan is also the most connected in December, although the national holiday does not occur in this month.

### Routes of travel

**Fig. 3:**
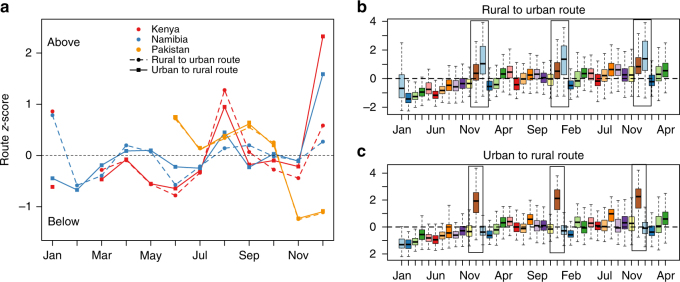
The relative amount of travel for each route per month. **a** For each route (i.e., an ordered pair of districts) within each country, a *z*-score was used to characterize deviations in travel across the year. Deviations were grouped by types of route (rural to urban (dashed line, circle), vs. urban to rural (solid line, square)); and associated median *z*-scores across the year are plotted (*y*-axis) for each month (*x*-axis). Median *z*-scores above 0 (dotted line) indicate travel volumes greater than baseline on that type of route; values below 0 indicate reduced travel volumes on that type of route (see Supplementary Information for remaining routes, rural to rural, and urban to urban). Kenya (red) and Namibia (blue) experience greater travel volumes between rural and urban districts in December in both directions; however, in January, travel from rural to urban districts is amplified in both countries, but the volume of travel from urban to rural districts is reduced. By contrast, Pakistan (orange) shows no strong discrepancies in directionality over the year, although overall volume of travel is high in December (Fig. 1) this is driven by individuals moving along routes that link pairs of urban, or pairs of rural districts (Supplementary Information). Data from Namibia allow us to evaluate the consistency of these patterns through time (time series starts at January 2011 until April 2014). Each box plot represents the distribution of district level route *z*-scores, again classified into types of routes, i.e., **b** rural to urban or **c** urban to rural. Seasonal trends remain consistent across years for the majority of routes, particularly the December and January differences (highlighted by the box)

Although the ratio of travel (or amount of travel to other districts relative to the amount of individuals who stayed in the same district), varied consistently across spatial scales in all countries (Fig. 3), regional spread could also be modified by fluctuations in the importance of particular routes (i.e., directionality of travel). To evaluate this, we categorized origins and destination districts of all routes as either urban (based on the proportion of the population living in urban areas in each district, see Methods) or rural (likewise). Four types of routes are then possible: urban to urban trips, urban to rural trips, rural to urban trips, and rural to rural trips (Methods). This classification yields two urban districts in Namibia, two in Kenya, and three in Pakistan; the majority of routes in all three countries are consequently classified as rural to rural (Supplementary Note 2; Supplementary Tables 2, 3).

To characterize fluctuations over the course of the year for each separate route (identified as an ordered pair of districts), we defined the *z*-score of the total amount of travel for that particular route in each month. We then grouped routes according to their classification (urban to rural, etc.) and compared median deviations for each type of route in each month of the year (Supplementary Note 2; Supplementary Figs. 5–10). Travel along all types of routes increases seasonally with national holidays and school terms (Fig. 3a), in line with country-wide analyses. However, the relative magnitude of changes in travel along specific types of routes varies according to season. Travel between urban and rural areas in both directions (Fig. 3b; Supplementary Information) is increased in December for Kenya and Namibia, but with a much greater relative increase for travel from urban to rural districts. Conversely travel from rural to urban areas is increased in January, and travel from urban to rural areas is reduced relative to baseline. We find similar qualitative results if districts are classified by income levels (Supplementary Note 2; Supplementary Figs. 11–14). This aligns with anecdotal evidence of individuals traveling from cities to their family homes in rural areas for the major holiday^42^. Results in Pakistan show a smaller difference between the types of routes (i.e., similar magnitudes for urban to rural and rural to urban trips over the course of the year), which may be related to the consistently large amount of travel, or incompleteness of data in this setting.

### Seasonal connectivity and disease spread simulation

How will seasonal differences in routes of travel affect the spread of a novel pathogen (beyond the effects of fluctuations of magnitude of travel)? We used a spatial diffusion model (Methods) to evaluate the general consequences of seasonal and spatial variation in connectivity (see Supplementary Note 3 and Supplementary Figs. 15–17 for an additional simulation). To reveal the impacts of mobility (rather than e.g., duration of stay), we focused on an array of acute pathogen life histories, leveraging an existing framing of the hazard of pathogen introduction^43^ for acute infections (Methods). We explored how the magnitude of transmission (from $\beta = 2$ to $\beta = 15$) shaped the lag before pathogen introduction into different districts under the connectivity (and thus directionality) settings defined by different months of the year in Namibia (since we had multiple years with which the impact could be compared). Even under high transmission, pathogen spread cannot occur in the absence of susceptible individuals, so we also varied the magnitude of susceptibility, across a range from 10 to 90% reflecting a low vs. high susceptibility setting. Many pathogens also show some degree of seasonal fluctuation in transmission (reflecting climatic forces or human aggregation^44^); but since our interest was in titrating the impact of mobility alone, we assumed a constant magnitude of transmission for these simulations. We focused on introduction into the capital city (Windhoek), as its greater international connectivity may make it a more likely origin of pathogen introductions into the country; and as analogy with other settings suggest that it might also be a core driver of endemic dynamics^23,45^.

**Fig. 4:**
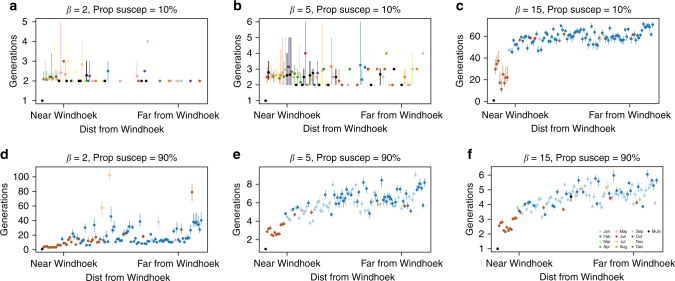
Consequences of seasonal travel in a spatial diffusion model. We simulated spatial spread of a pathogen (Methods) starting in the most populated district (Windhoek East in Namibia). Pathogen transmissibility, proportion of the population susceptible, and monthly connectivity value between districts defined a time varying hazard of introduction into each district. We compared the time (*y*-axis in days) each location became infected vs. Euclidean distance from Windhoek (*x*-axis). Each district is colored by the slowest month (i.e., when the pathogen on average took the longest to reach a particular district). To explore the impact of different pathogen life histories, we compared varying magnitudes of transmission (*β*) ranging from low (**a**, **d**) ($\beta = 2$) to intermediate (**b**, **e**) ($\beta = 10$) to high (**c**, **f**) ($\beta = 15$). We compared across two proportion of the population susceptible reflecting **a**–**c** low (10%) and **d**–**f** high (90%) values. The bars show the range in timings across the different months. We performed each hazard simulation 100 times per month and then average across simulations and months. When a small percentage of the population is susceptible and transmission is low (**a**), many locations will not become infected. Those that are infected are infected early, likely related to stochastic nature of the simulations. In medium–high proportion of the population susceptible simulations, for places nearby Windhoek, the time they become infected is consistent with physical distance, i.e., those closest to Windhoek become infected earlier. The only exception is the influenza-like simulation (e.g., corresponding to low magnitude of transmission), where many locations do not become infected; and this pattern is intensified where susceptibility is low. As the transmission parameter increases, the pathogen spreads faster throughout the population, often reaching all districts within a few generation times (i.e., **e**,** f**). The timing at which a district becomes infected will also depend on when during the year the pathogen was introduced, given seasonal fluctuations in mobility. For locations nearby Windhoek, the pathogen reaches these locations the latest in December, reflective of the decrease in travel from Windhoek East to nearby locations in this month

For pathogens with sufficiently high transmissibility, we found a classic signature of distance decay for the timing of pathogen introduction: introduction occurred later in districts further from Windhoek. Higher transmissibility also results in more rapid spatial spread (Fig. 4; Supplementary Figs. 18, 19). For pathogens with weak transmission, both seasonal patterns and the impact of distance are obscured; further, many locations escape infection in this scenario (86%). Those locations that become infected, often become infected early in the simulation, although the range of estimates varies greatly.

In the context of our specific aim of titrating the impact of seasonal differences in mobility on the spatial spread of an infectious disease, we find differences in spread associated with time of year of pathogen introduction. Indeed, the overall speed of pathogen spread depends not only on characteristics of the pathogen, but also the month of its introduction into the country. For each district, we identified the introduction time corresponding to the location becoming infected with the greatest delay. Districts closest to Windhoek, although infected early relative to other districts, are infected with the greatest delay for introductions occurring in December. This is in contrast to all other districts: the greatest delay before infection is associated with introductions occurring in either January or February for a wide range of parameter values (Fig. 4). This result is driven by seasonal fluctuations across particular routes of travel alone, since we are not evaluating the role of magnitude of travel here.

## Discussion

Human mobility is seasonal. Here, we used a unique data source to reveal the magnitude and consistency of temporally varying human mobility across spatial scales. We identify social and cultural drivers as having a key role in shaping these patterns, an effect which shows marked consistency both within and between countries. Although we were unable to test the repeatability of temporally varying trends in Pakistan and Kenya, in Namibia, seasonal patterns were highly repeatable. An important consequence of this phenomenon is that countries are most connected during national holidays.

Investigation of fluxes along specific routes reveals further nuance: directionality of mobility also changes over the course of a year. In Kenya and Namibia, travel from urban to rural areas increases in December and January, and travel from rural areas to urban areas in January, suggesting travel from urban areas for Christmas and back after the holidays. This directionality also affects spread of an introduced pathogen. Our model of geographic spread combined with a connectivity matrix reflecting each month for which data was available revealed geographical and temporal differences in when a location may become “infected” as a result of differences in directionality of travel at different times of year. The consistency of the differences we obtain across a diversity of pathogen life histories suggest that there may be some potential to leverage urban/rural directionality in planning timing of control effort deployment across districts; and that this will be of greatest utility for high transmission pathogens. For less transmissible pathogens, the patterns of spread are less predictable. However, the spatial model of pathogen spread indicates that this can shape the spread of infectious disease, which will in turn shape the best timing for investments in efforts toward the control of pathogens such as malaria^5^.

Moving up to the national scale, all three countries are the most connected in December, a time when seasonal travel fluxes are highest (although note previous caveats about the scale of data in Pakistan). These results may have implications for pathogens emergence and global pandemics—homogenization of the timing of holidays across large regions of the globe will make these areas ripe for rapid pathogen spread, by maximizing connectivity at particular times of year. Overall, we uncover consistent differences in patterns of pathogen spread as a result of seasonal travel (with Kenya and Namibia the most similar), suggesting generalizable trends in mobility may be identifiable and are likely to have considerable impacts on the landscape for national and international infectious disease spread.

Ultimately, building models that combine robust estimates of seasonal travel with pathogen biology and data on disease incidence has the potential to define the best timing and spatial distribution of control efforts over the course of a year (although much of the nuance of other drivers of seasonality will have to be addressed^30^). This is one of many threads that could contribute to updating the current simple public health schema where both burden and control efforts are evaluated as yearly aggregates^29^. However, no model is better than its underlying data. Mobile phone data are likely to provide an incomplete picture of seasonal travel for a number of reasons. Mobile phone subscribership, although increasing^46,47^, does not represent a randomized sample of the population. From previous work, we know that mobile phone ownership is biased toward more educated, urban males, a group that may be more mobile^46^, and potentially misses parts of the lowest income groups^48^. Thus, while the data may capture the full scope of travel of some individuals, the details of biases may be rather complex. Mobile phone data are also likely to under sample the movement of children, which is potentially problematic given their importance in the transmission of many pathogens^23^. Mobile phone coverage and mobile phone ownership may be low in particular locations (e.g., remote rural locations), limiting our understanding of the full spatial context; just as the extent of the time series we had available (particularly for Pakistan) limited our understanding of the full temporal scope. The higher amount of travel in Kenya and Pakistan compared to Namibia may in part be related to differential ownership of mobile phones, but might also reflect biases in the sources of data available (e.g., coverage within the population, etc.). Although we focused on differences in travel on a rural–urban gradient, using a measure of socio-economic status (Supplementary Information) that may better capture differential ownership patterns qualitatively show similar results (Supplementary Figs. 12–14). However, since there is no single proxy for socio-economic status, these results may vary depending on the method of categorizing locations.

As we were primarily interested in how travel patterns changed over the course of the year, differential ownership should not too greatly bias our results (unless there is considerable hidden spatial heterogeneity in seasonality that deviates from the broad patterns identified here). In both Kenya and Namibia, the mobile phone operator had the largest market share at the time of data collection implying that these samples are likely reflective of the majority of mobile phone owners, and thus likely have reasonable spatial representation. Although the percentage of the population included from Pakistan was lower (22%) than the other countries, this could partially be explained by multiple operators in the country limiting the subscriber base of any individual operator. However, it is unlikely we are completely missing more remote areas since the operator has a higher than expected coverage in these regions^2^.

Although this analysis improves upon yearly aggregates of mobility, we still chose to aggregate movement to a daily time step. Small-temporal scale commuting behavior within a single day will consequently be missed, despite its potential importance for transmission of pathogens such as influenza^49^. Likewise, long-term migration trends that are commonly observed in the national census may be missed by the duration of these data sets, although the relative patterns may be similar^50^. Previous work suggests that although the magnitude of travel obtained from the census does not match the definition of travel we use from mobile phone data, the relative ranking of routes of travel remains the same, so that overall trends in long-term migration may be captured^51^. Finally, our analysis does not include cross-border and international travel, which may also be seasonal^51^, and likely to interact in complex ways with pathogen introduction. Without a firm understanding of cross-border travel in these countries and the transmission characteristics of a specific pathogen, it is unclear how this will modulate the outcomes we have described. We believe that, particularly for the emergence of novel pathogens, cross-border movement will play an important role in the location of the first introduced case into these countries, which will impact the spatial diffusion patterns.

Our analysis provides a comparative assessment of seasonal fluctuations in directionality of travel, finding intriguingly similar trends across three different countries. Evidence from Namibia indicates that both magnitude of travel but also its directionality varies repeatedly across the year; data from all three countries points to consistency in patterns across spatial scales. Accumulating data from more countries will allow us to identify the degree to which there are generalizable patterns of seasonal travel. This would open the way to investigating whether general patterns for infectious diseases and their movement across scales emerges, grounding this line of research in both knowledge of key social and cultural events, but also the importance of broad structural features, such as urban vs. rural communities. Building toward an ability to predict the consistency and/or variability of introduction times of pathogens into different locations across the year will broadly contribute to epidemic or pandemic preparedness.

## Methods

### Geographic data

For each country, district and province level shape files and areas were obtained from GISDiva.org. The population of each country, province, and district was calculated using 100 × 100 m population distribution data sets from WorldPop (Supplementary Fig. 1). Although we focused on dynamic population movements, these static estimates are based on the census values that represent a single population estimate from the date of the census. We classified districts as either urban or rural based on the census reported percentage of the population considered urban; districts where at least 25% of the population was considered urban were classified as urban districts. All others were classified as rural districts.

### Quantifying mobility patterns from mobile phone data

We analyzed mobility data extracted from mobile phone CDRs from operators in three countries (from a single operator per country): Kenya, Namibia, and Pakistan. These data vary in terms of the number of subscribers, time frame, and number of mobile phone towers (Table 1; Supplementary Fig. 1). The number of subscribers in each data set and the number of mobile phone towers both varies by an order of magnitude between countries (Table 1). However, they all have similar geographic mobile phone tower coverage in terms of the percentage of the country serviced by mobile phone towers (Supplementary Fig. 1) and district areas (Supplementary Fig. 20). Mobile phone ownership varies between countries and may be due to mobile phone adoption and the number of mobile phone operators in each country. For example, in Pakistan we analyzed data from ~40 M subscribers in a country of ~182 M (~20% of the population), however there are a number of mobile phone operators in the country, which likely reduce the market share of any single company. In contrast, the data analyzed in Namibia, a country with few mobile phone operators, included near 1.8 M subscribers in a country of ~2.3 M (~78% of the population). Operator market share may be spatially heterogeneous; however, subnational market share data are not available in many low- and middle-income contexts.

To compare across countries and contexts, mobility patterns from each data set were extracted using the same method. Briefly, we assigned each individual subscriber a primary daily location based on either the most frequently used mobile phone tower or the most recently used mobile phone tower if a call was not placed on the day. In the Kenya and Pakistan data, each subscriber is assigned a location for each day in the data set; regardless of the first day they made a call/SMS. However, given the long time frame of the Namibia data set and the changes in the number of subscribers over this time (~970,000 subscribers in 2010 vs. 2,200,000 subscribers in 2014, see Supplementary Fig. 21), we estimated the primary daily location for subscribers only for the period between their first and last call record, to avoid overestimation of stays. We aggregated daily tower locations to administrative level 2 (referred to as a district) in each country. Based on each subscriber’s district location on consecutive days, we calculated the number of trips between all districts. Here a trip between districts A and B on day *N* is measured if a subscriber's daily location is district A on day *N* and district B on day *N* + 1.

We aggregated the number of trips between all pairs of districts for each day in each data set and compared the number of trips between districts to the number of stationary individuals who have not changed their district location on consecutive days. Using these two values, we defined the magnitude of travel as the ratio of the subscribers that traveled on consecutive days. As the spatial and population scales in all three countries varied, using the ratio of the population traveling provides a tractable and roughly comparable unit of measure (Supplementary Fig. 20).

### Calculating country-wide mobility patterns

For each country, we calculated the *z*-score of the raw ratio of travel values per location per day (Fig. 1). The overall country-wide trend is shown (in black) and is the daily median value smoothed using a spline.

### Drivers of seasonal travel

To identify overall drivers of seasonal travel, we analyzed the ability of social (school terms and major national holidays) and climatic variables (rainfall and temperature) to predict travel values. In each country, we identified the date of the major national holiday (Christmas on 25 December in Kenya and Namibia, Ramadan between 9 July–7 August 2013 in Pakistan). We used the national average school term breaks for each country. School break time in Kenya and Namibia varied by year, but in general were during April, August, and December/January. Pakistan has a single long break from July–August. We also calculated the monthly average temperature and monthly total precipitation per district based on the nearest weather station (weather station data obtained from www.ncdc.noaa.gov). We also used a location variable, which was a factor variable for each district.

We performed a lasso regression to predict the monthly average ratio of travel per district using school terms, temperature, precipitation, a location factor, and a major holiday indicator (Supplementary Information).

### Routes of travel

We classified each district as either urban or rural based on the percentage of the population living in urban areas. Districts were considered urban if at least 25% of the population lived in an urban area. This threshold was chosen to ensure that all countries had at least one urban district (Supplementary Table 2). We classified each route of travel based on the urban/rural classification of the origin and destination districts (i.e., urban to urban, urban to rural, rural to urban, and rural to rural, see Supplementary Table 3). We then analyzed each route of travel separate and calculated the relative seasonal fluctuations in travel using a *z*-score of the number of trips. We then grouped routes based on the urban/rural classifications (see Supplementary Information, Supplementary Table 2, Supplementary Figs. 5, 6 for the results using various cut off points in Namibia).

### Simulating outbreak risks with time-varying travel patterns

We also simulated a spatial spread model that defined the outbreak risk in each location per month. In each district, a new outbreak will spark if there is contact with an infected individual from elsewhere and both the size of the susceptible population and magnitude of transmission will enable an outbreak^4^. This can be reformulated as a time-varying hazard:

1 $$h\left( {t,j} \right) = \frac{{\beta S_{t,j}\left( {1 - {\mathrm{exp}}\left( { - \sum _kc_{j,k}x_{t,k}} \right)\left. {S_{t,j}} \right)} \right)}}{{1 + \beta S_{t,j}}}$$

where *β* reflects the magnitude of transmission; $S_{t,j}$ is the proportion of the population in location *j* at time *t* that is susceptible; $c_{j,k}$ reflects mobility from location *k* to location *j*; and $x_{t,k}$ is the proportion of the population at time *t* in location *k* that is infected. We set a fixed starting proportion of the population susceptible ($S_{t,j}$) across all locations that we varied for each simulation (Supplementary Information; Fig. 4). We did not include a seasonally varying transmission value and chose a fixed value of *β* per simulation reflecting *R*_0_ estimates for various pathogens. The extension to include a seasonally varying transmission parameter would be trivial to include in the simulation, but would require an assumed relationship between seasonal transmission and connectivity. We compared the simulations using different connectivity matrices $c_{j,k}$ per month where travel was normalized to understand the changes in routes of travel as opposed to the magnitude of trips. We also took the simplifying assumption that transmission does not vary spatially.

We then simulated a stochastic process of introductions into districts using the above hazard:

2 $$I_{t + 1,j}\sim {\rm{Binom}}(h\left( {t,j} \right))\;{\mathrm{for}} \; I_t = 0$$

In locations where the disease has been introduced, we simulated deterministic dynamics including both transmission and susceptible depletion according to:

3 $$I_{t + 1,j} = \beta S_{t,j}I_{t,j}\,{\mathrm{for}}\,I_t \,  >\, 0$$

4 $$S_{t + 1,j} = S_{t,j} - I_{t,j + 1} + b$$

with a fixed approximate birth rate, *b*, (~29 per 1000 per year)^52^. Here, the generation time is implicitly included in the discrete model since it assumes that infections are cleared within a single time step.

### Code availability

R code to perform the infectious disease simulation is available from the corresponding author (A.W.) upon request.

### Data availability

The data that support the findings of this study are not publicly available since that would compromise the agreement with the mobile phone operators that made the said data available for research. Information about the process of requesting access to the data that support the findings of this study can be obtained from the corresponding author (A.W.).

## Electronic supplementary material

Supplementary Information
