# Supplementary material for: Multinational patterns of seasonal asymmetry in human movement influence infectious disease dynamics
Source: Nat Commun. 2017 Dec 12;8:2069. doi: 10.1038/s41467-017-02064-4 (PMC5727034; doi:10.1038/s41467-017-02064-4)
Supplement: Supplementary file 1 — Supplementary Information [file 41467_2017_2064_MOESM1_ESM.pdf]

Supplementary Information

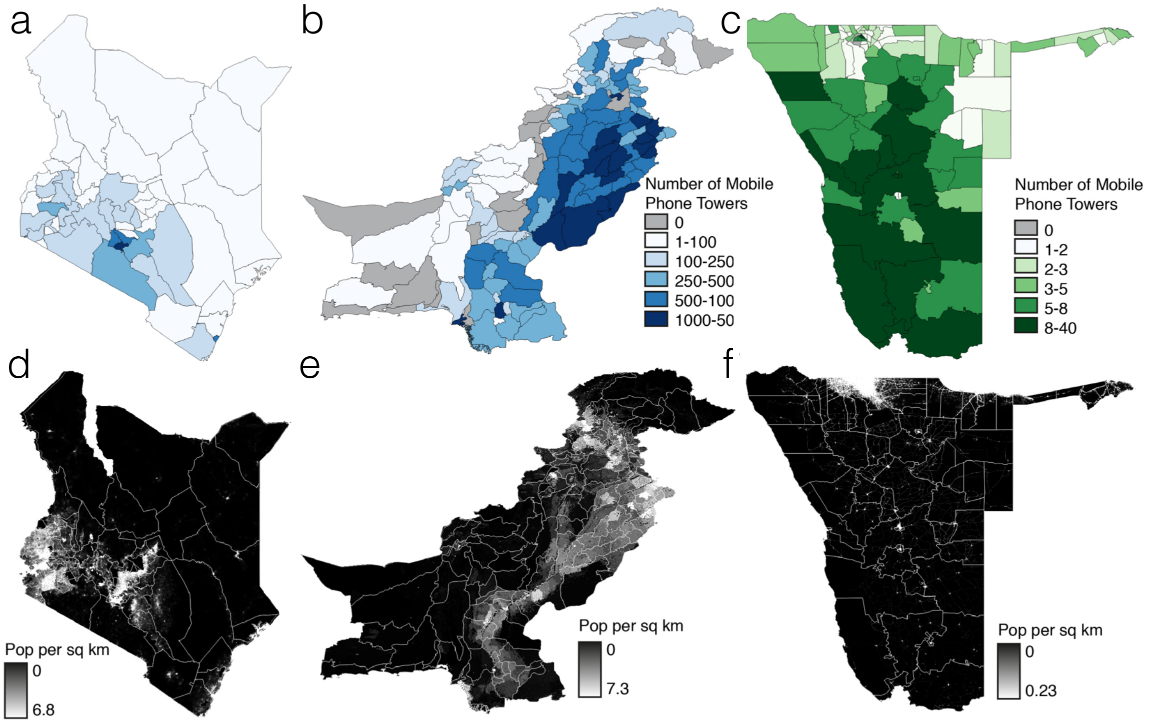

Supplementary Figure 1: The mobile phone coverage and general travel patterns for each country. We analyzed mobility patterns extracted from mobile phone data for a) Kenya, b) Pakistan, and c) Namibia. These data were provided by various mobile phone providers (see Materials and Methods) and encompass mobility aggregated from call data records for subscribers over various time frames. In general, population density d) Kenya, e) Pakistan, and f) Namibia (show in white – grey) correlates with tower coverage (areas shown in grey, see Materials and Methods). These data were obtained over different time periods with the earliest from Kenya (2008-2009) and most recent and longest from Namibia (2010-2014). Using these data, we analyzed mobility patterns based on travel between administrative level 2 areas. District boundaries were obtained from: www.diva-gis.org (a – f), population data (d, e, f) was obtained from: www.worldpop.org.uk and were created using ArcGIS 10.3.

Supplementary Table 1: The average monthly percentage of the population who have traveled. We calculated the total percentage of trips between districts for consecutive days. Using these country-wide values, we calculated the average percentage of travel per month.

	Kenya data*	Pakistan data	Namibia data 2012	Namibia data 2013	Namibia data 2014
Jan	5.03		15.05	12.87	12.45
Feb			15.20	12.89	12.66
Mar	5.17		15.21	13.00	13.13
Apr	5.24		15.22	13.22	12.90
May	5.32		15.35	13.36	13.16
Jun	4.57 (2008)<br>5.24 (2009)	34.02	14.77	12.46	12.57
Jul	4.78	32.48	14.66	12.77	12.94
Aug	5.08	32.71	15.13	13.12	13.37
Sep	5.08	33.02	14.86	13.11	13.10
Oct	5.02	33.44	15.03	13.15	13.02
Nov	5.29	34.27	14.20	12.86	12.95
Dec	5.95	34.51	14.35	13.79	13.52
* Note: January – June data are from 2009, June – December data are from 2008.					

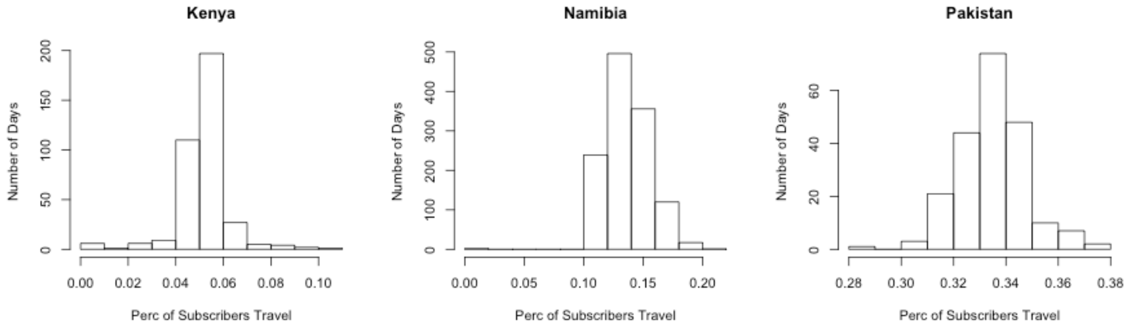

Supplementary Figure 2: The distribution of percentage of subscribers who travel between districts per day.

Supplementary Note 1: ***Variability in space and additional drivers***

To identify how consistent these general patterns were on finer scales, we next investigated the travel patterns on a finer spatial scale to identify if there exists heterogeneity on small administrative units. We aggregated district-level travel patterns to the province (administrative level 1) and country scale based on the location of each district. In all three countries, the province level seasonal travel patterns were similar to the country-level pattern (mean Pearson's correlation coefficient for Kenya: 0.90, Namibia: 0.78, Pakistan: 0.95). The relationship was the strongest in Pakistan and weakest in Namibia.

To investigate the relationship further, we compared the travel percentage time series of a province with the time series for all districts within the province and calculated the correlation for each pair between district level and the province level travel. Overall, district level travel patterns are correlated with the province level pattern, although this relationship varies geographically and by country. In Pakistan, the majority of districts' travel patterns were similar to the province average travel pattern (see Supplementary Fig. 3). In contrast, the weakest correlations were in Namibia and in all three countries, the most rural areas of the countries tended to have weaker relationships (see Supplementary Fig. 3). These results suggest that seasonal travel is fairly consistent within a single country on various spatial scales; discrepancies can be attributed to statistical fluctuations.

To analyze the relationship between travel volumes and climatic drivers, we first compared seasonal travel values from each country with two climatic variables: temperature and precipitation. For each country, we analyzed temperature and precipitation values measured at weather stations (www.ncdc.noaa.gov) during the time period of each data set. For each district, the mean of all weather stations' monthly temperature (deg C) and total amount of precipitation (mm) were calculated using all of the weather stations in each district. For districts that did not have a weather station, we used the values from the nearest (using Euclidean distance) weather station. The climate data shows remarkable temporal and spatial variation within each country. Of the three countries, Pakistan has the most extreme climate with large geographic and temporal differences in both temperature and precipitation. In general, there are four distinct seasons in the country: dry/cool, dry/hot, monsoon, and light rains. These temperatures can range between 12C-35C (monthly mean range over the data set, 13-33C). Namibia has a more temperate climate with two rainy seasons (long rains often between February – April, short rains between September – November) although the rainfall patterns have increasingly become more variable with droughts common. Kenya's climate ranges from tropical along the coast to mountainous terrain in the highlands.

We compared these climatic variables with average monthly travel volume in each country. Based on anecdotal evidence, we would expect travel to have a negative relationship with precipitation values. During months of peak rainfall, non-paved roads may be impassable in very rural areas reducing travel. In Pakistan we found very little evidence that seasonal changes in travel are related to either climate variable (see Supplementary Fig. 3). In all three countries, the relationship with precipitation may be affected by poor precipitation data collection.

In Kenya, all travel is positively correlated with temperature (more travel during hotter seasons) with the strongest relationships in central Kenya (see Supplementary Fig. 3). Surprisingly travel was positively correlated with precipitation (more travel during rainier seasons) in central Kenya with a negative relationship found along the coast and in western Kenya.

The clearest relationship between climate and travel is in Namibia. We found a linear relationship between the correlation between travel and precipitation and temperature. Districts with a strong positive relationship between travel and temperature also had a strong positive relationship between travel and precipitation (and vice versa). The areas of the country that had a negative relationship between travel and climate (less travel during hotter temperatures or more precipitation) were often less populated districts.

Unlike Kenya, there is no clear spatial difference between what parts of the country have a positive or negative correlation between climate and travel (see Supplementary Fig. 3). In Pakistan, there are no clear trends between climate and travel. There does not appear to be any clear geographic pattern, although the limited timeframe of the data may make any signal difficult to detect. Overall, travel between districts in Kenya and Namibia was negatively correlated with school terms implying that greater amounts of travel occurred during school breaks.

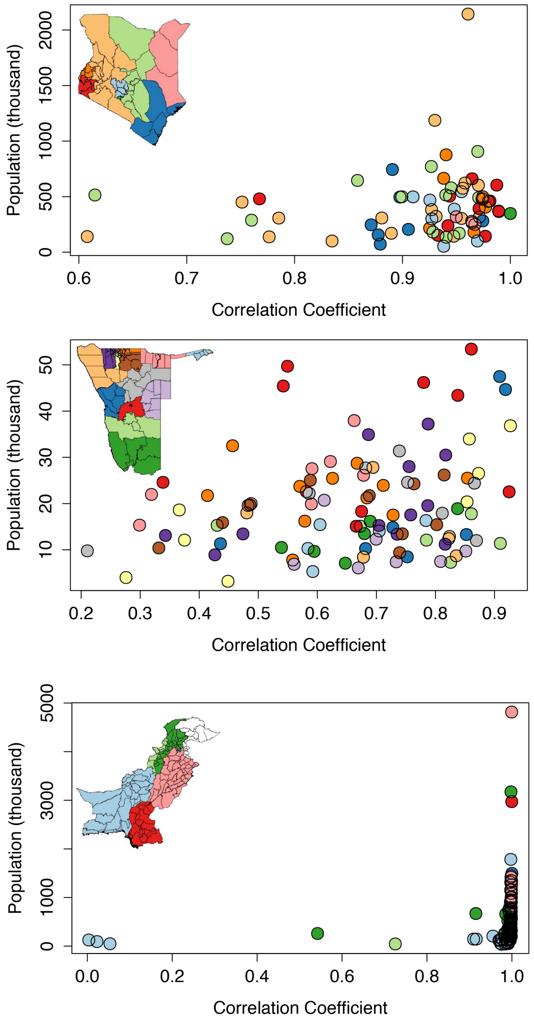

Supplementary Figure 3: The relationship between population and the correlation between province and district level travel patterns. For each district, we compared the percentage of subscribers traveling to other districts to the province level pattern of the percentage of subscribers traveling to other provinces. The Pearson's correlation coefficient between the district and corresponding province level travel is shown versus the population of the district. Each district is colored by the province. In general, there is a weak relationship between the correlation and the population. District boundaries were obtained from: www.diva-gis.org and were created using ArcGIS 10.3.

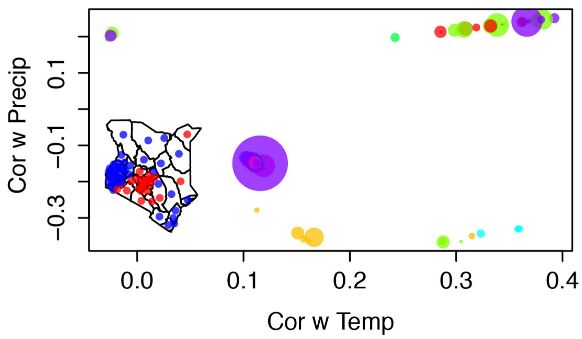

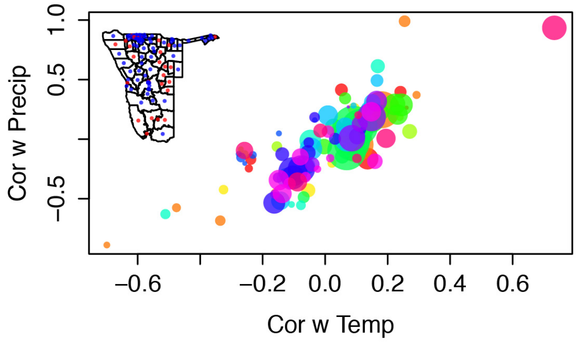

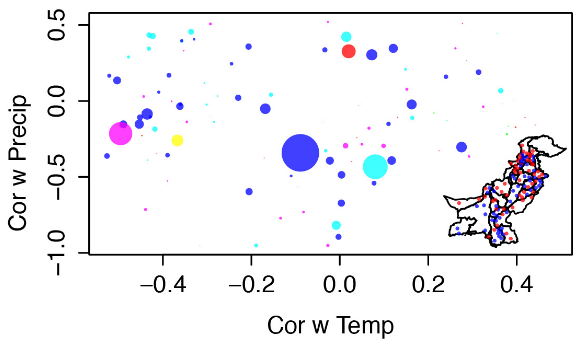

Supplementary Figure 4: We calculated the district level correlation between the monthly average percentage of subscribers traveling to other districts and the corresponding monthly average temperature and monthly total precipitation. The values for Kenya (top), Namibia (middle), and Pakistan (bottom) are shown. In each map, districts are colored red if the relationship was negative, blue if the relationship is positive. The color of the points corresponds to the corresponding province. The correlation values are the strongest in Namibia with weaker relationships in Kenya and Pakistan. In Kenya travel volumes for the majority of places are positively correlated with temperature (higher temperatures ~ higher percentage of travel). This is untrue in both Namibia and Pakistan. In Namibia, places that are strongly correlated with temperature are also strongly correlated with precipitation possibly due to the stronger relationship between temperature and precipitation. There does not appear to be a systematic relationship between correlations with

temperature and precipitation in Pakistan. District boundaries were obtained from: www.diva-gis.org and were created using ArcGIS 10.3.

We tested the role of social drivers (school terms, national holidays) versus climatic drivers for monthly values. If the majority of the days in the month were during a school break, we assumed that month counted as a non-school time. We performed a stepwise linear regression to predict the monthly average percentage of travelers per district using school terms, temperature, precipitation, a location indicator, and a major holiday indicator.

Supplementary Note 2: Differences in travel patterns based on income bracket and percentage of the population urban

Supplementary Table 2: The number of districts classified urban in each country. For each country, we calculated the percentage of the population considered urban in each district (see Materials and Methods). In the main text, we used the 25% threshold to classify districts, i.e. if at least 25% of the population was considered urban then the district would be classified urban. Using different thresholds, the number of districts considered urban in each location varied.

**Threshold - percentage of the population considered urban**	**Number of districts classified urban - Namibia**	**Number of districts classified urban - Kenya**	**Number of districts classified urban - Pakistan**
5	5	6	8
10	3	2	4
15	3	2	4
20	2	2	3
25	2	2	3
30	2	1	3
35	2	1	3

Supplementary Table 3: The number and percentage of routes in each country classified by the percentage of the population in the origin and destination considered urban.

Country	Rural to Rural	Rural to Urban	Urban to Rural	Urban to Urban
Kenya	4489 (94.3%)	134 (2.8 %)	134 (2.8%)	4 (0.1%)
Namibia	10609 (96.2%)	206 (1.9%)	206 (1.9%)	4 (0.0%)
Pakistan	10000 (78.3%)	300 (2.3%)	300 (2.3%)	9 (0.01%)

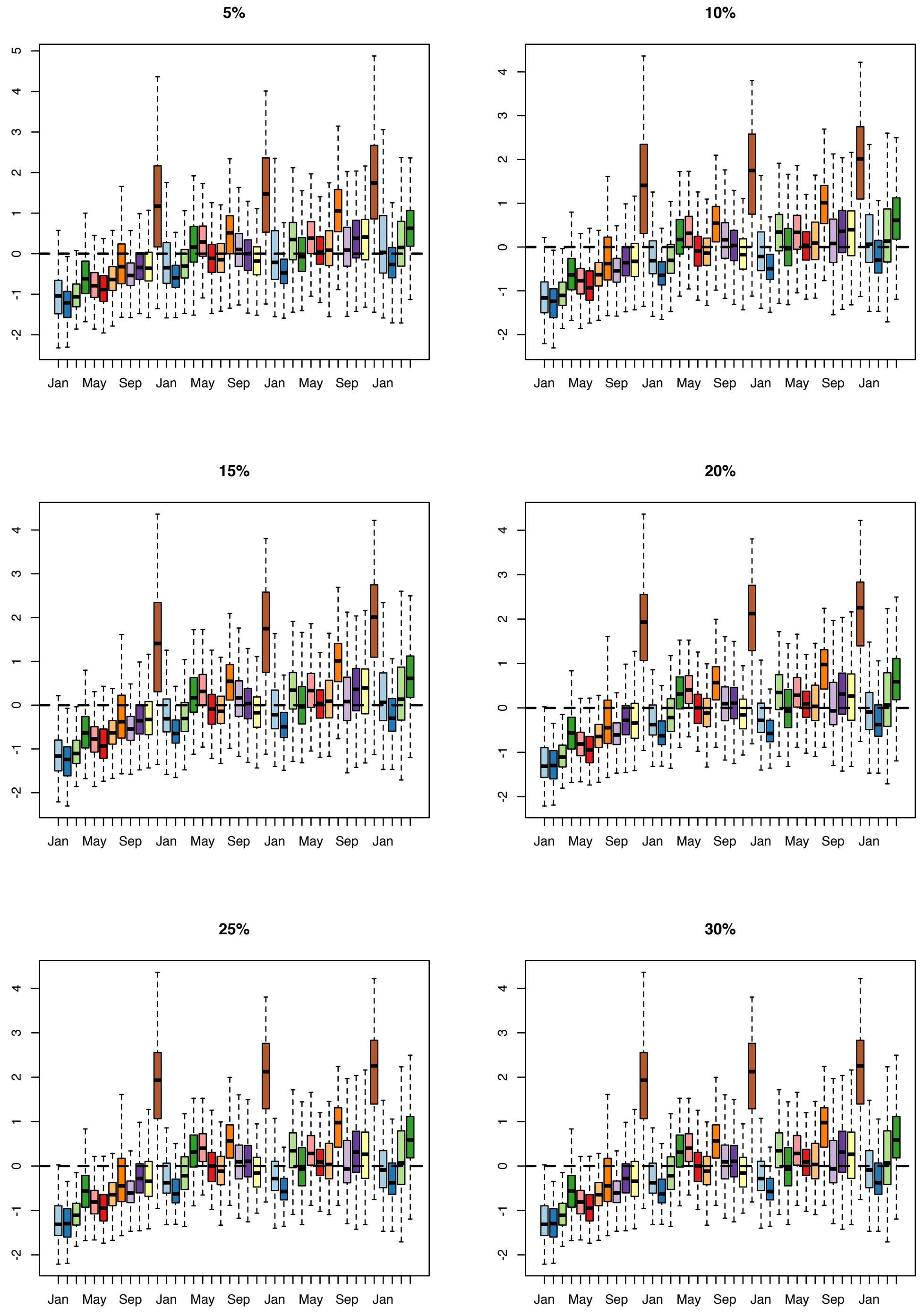

Supplementary Figure 5: Travel from rural to urban areas in Namibia. Data from Namibia allows us to evaluate the consistency of these patterns through time (time series starts at January 2011 until April 2014). Each boxplot represents the distribution of district level route z-scores for rural to urban travel. We varied the threshold used to classify a district as urban (labeled) and calculated the corresponding z-scores for urban to rural travel. Seasonal trends remain consistent across years for the majority of routes with peaks during December, regardless of the threshold.

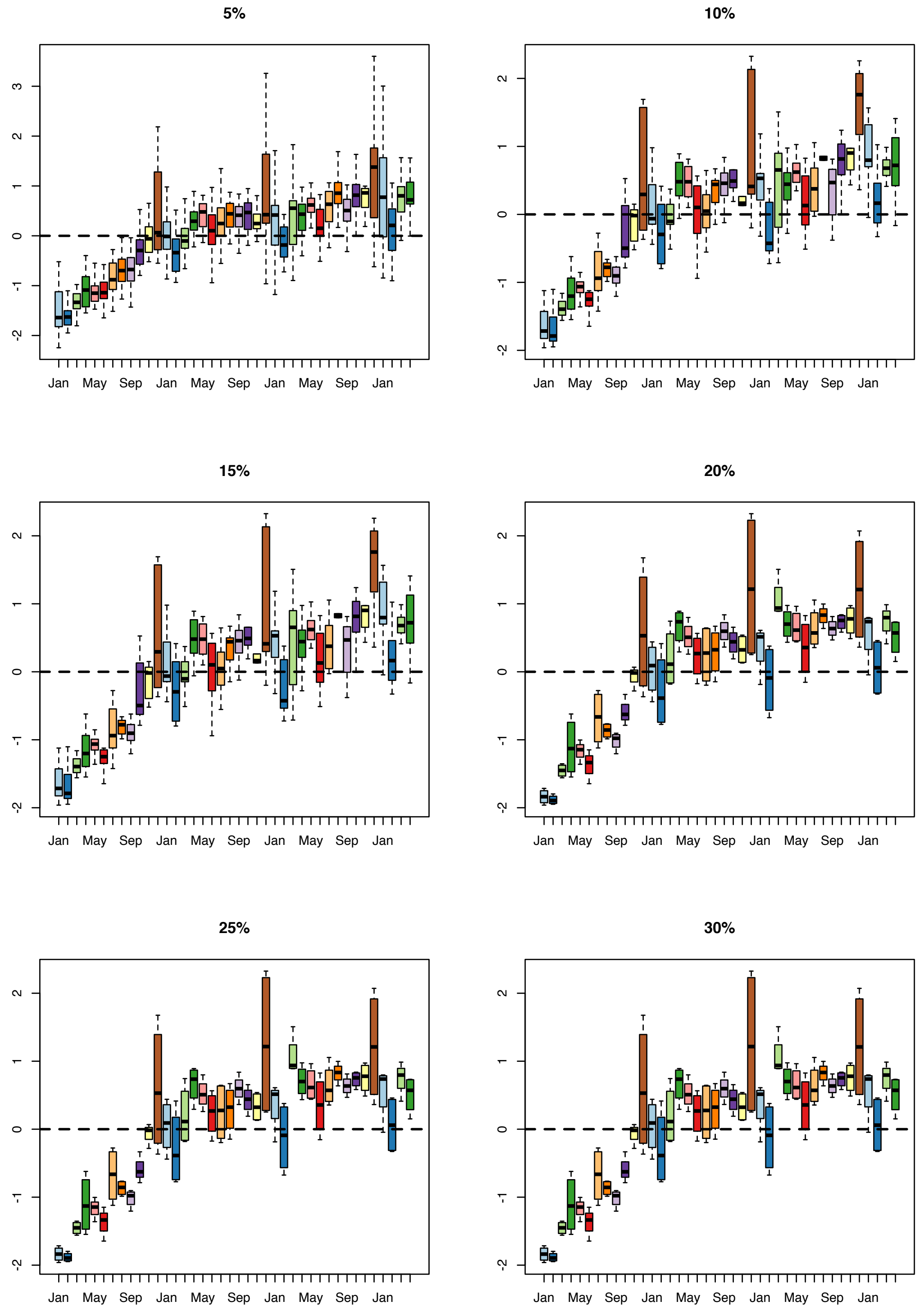

Supplementary Figure 6: Travel from urban to rural areas in Namibia. Data from Namibia allows us to evaluate the consistency of these patterns through time (time series starts at January 2011 until April 2014). Each boxplot represents the distribution of district level route z-scores for urban to rural travel. We varied the threshold used to classify a district as urban (labeled) and calculated the corresponding z-scores for urban to rural travel. Seasonal trends remain consistent across years for the majority of routes with peaks during December, regardless of the threshold.

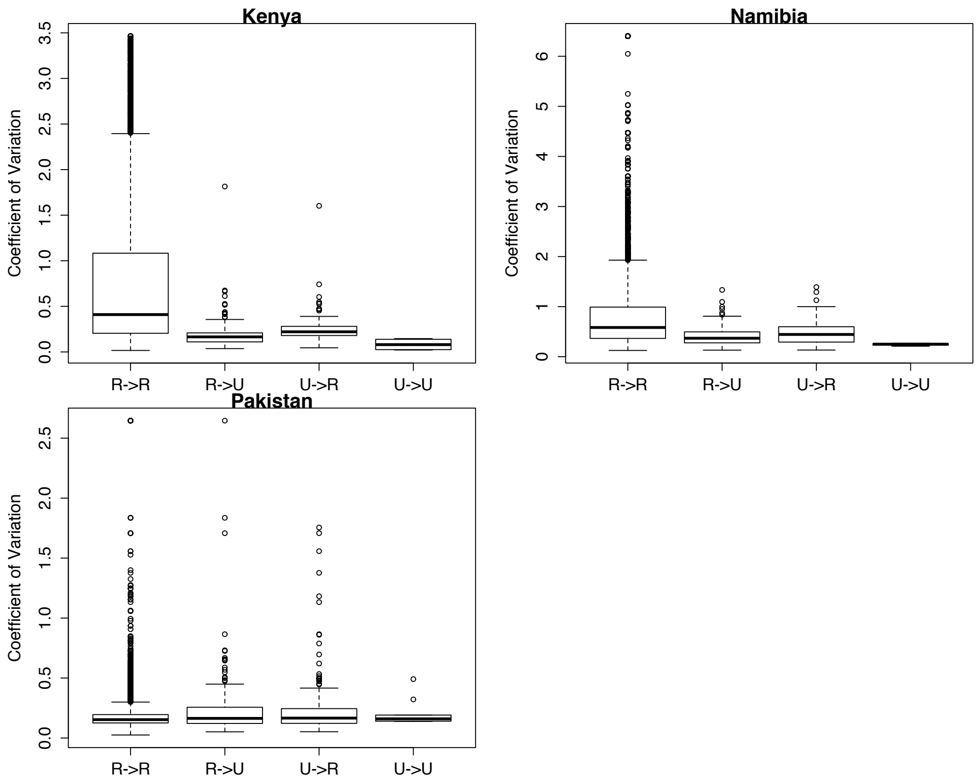

Supplementary Figure 7: The coefficient of variation for different routes of travel including Rural to Rural (R->R), Rural to Urban (R->U), Urban to Rural (U->R) and Urban to Urban (U->U) for all three countries.

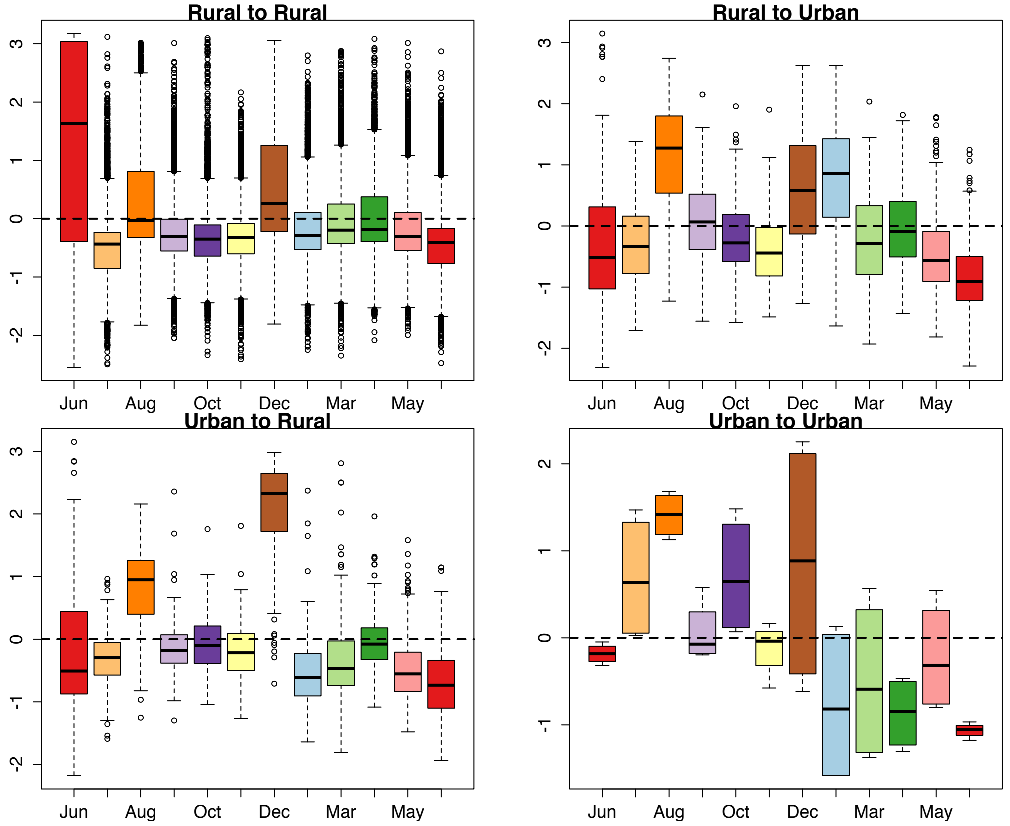

Supplementary Figure 8: The z-scores for different routes of travel per month in Kenya. For each route of travel between districts, the z-score of travel was calculated (see Materials and Methods). These routes were classified based on the percentage urban of the origin and destination district. The distribution of monthly z-scores is shown.

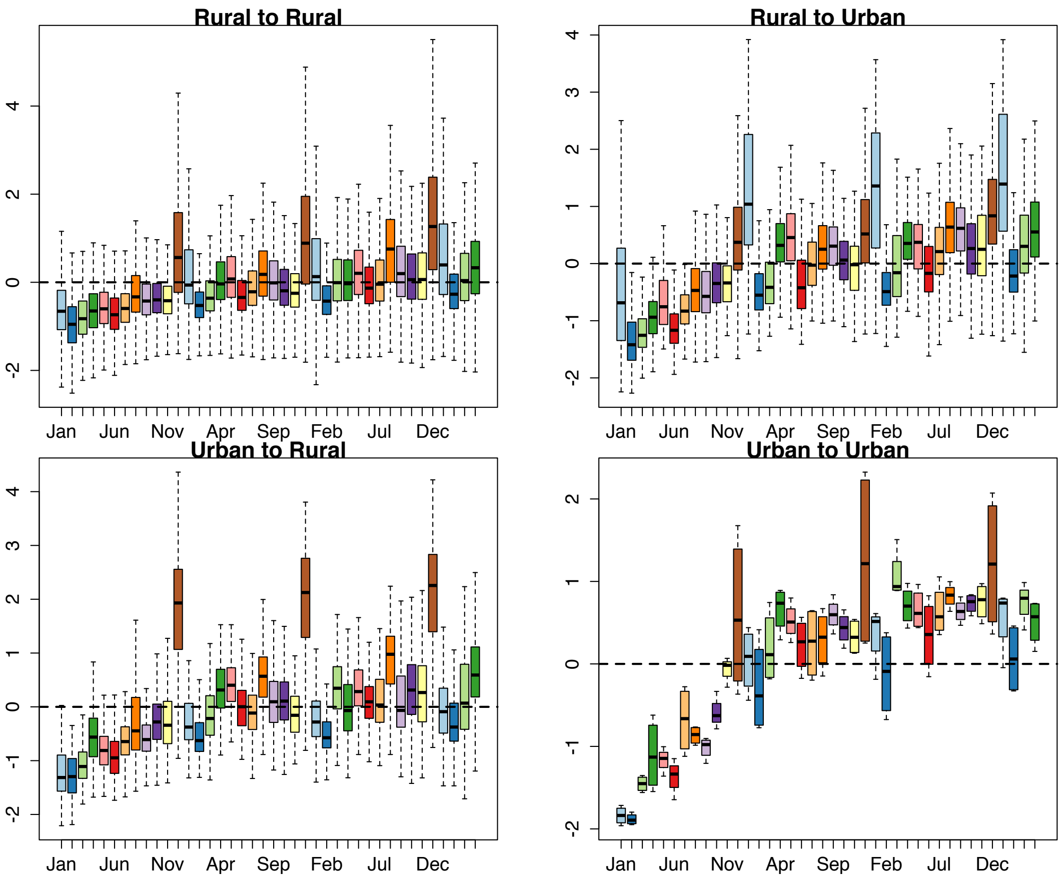

Supplementary Figure 9: The z-scores for different routes of travel per month in Namibia. For each route of travel between districts, the z-score of travel was calculated (see Materials and Methods). These routes were classified based on the percentage urban of the origin and destination district. The distribution of monthly z-scores is shown.

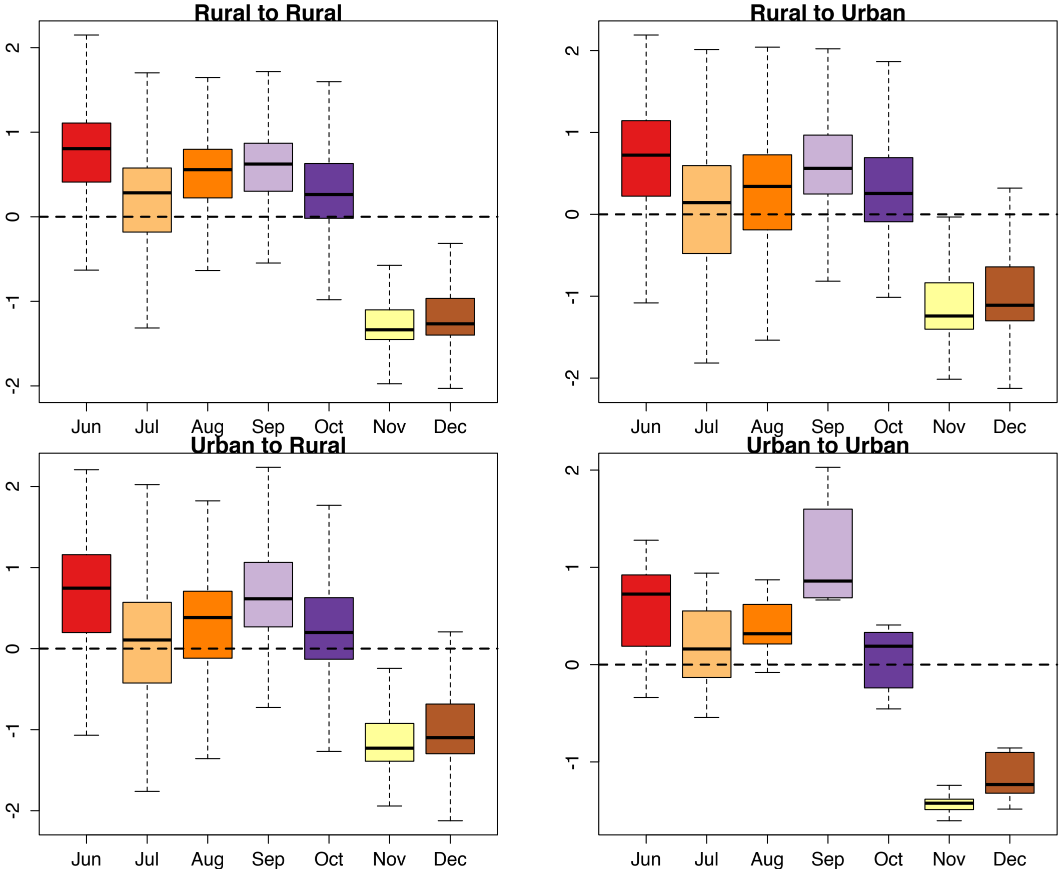

Supplementary Figure 10: The z-scores for different routes of travel per month in Pakistan. For each route of travel between districts, the z-score of travel was calculated (see Materials and Methods). These routes were classified based on the percentage urban of the origin and destination district. The distribution of monthly z-scores is shown.

High income versus low income travel patterns: The urban versus rural divide likely obscures between district socio-economic status heterogeneity. We utilized the Demographic Health Survey (DHS) data for all three countries as a proxy for socioeconomic status [1]. In each country, information on household assets is used to create an index that represents the wealth of households who were interviewed. This provides a measure each of the largest administrative units (normally a province) of the proportion of the province population in each wealth quintile. However, it is important to note that these results are relative to each country – i.e. low income in one country may not be comparably low income in another country – meaning it is not possible to compare these measures between countries.

For each country, we considered the percentage of the de jure population in the lowest wealth quintile. A location was considered low income if at least 25% of the population was in the lowest wealth quintile, otherwise high income (see Supplementary Fig. 11). We then compared the results between travel patterns from/to high-low income provinces.

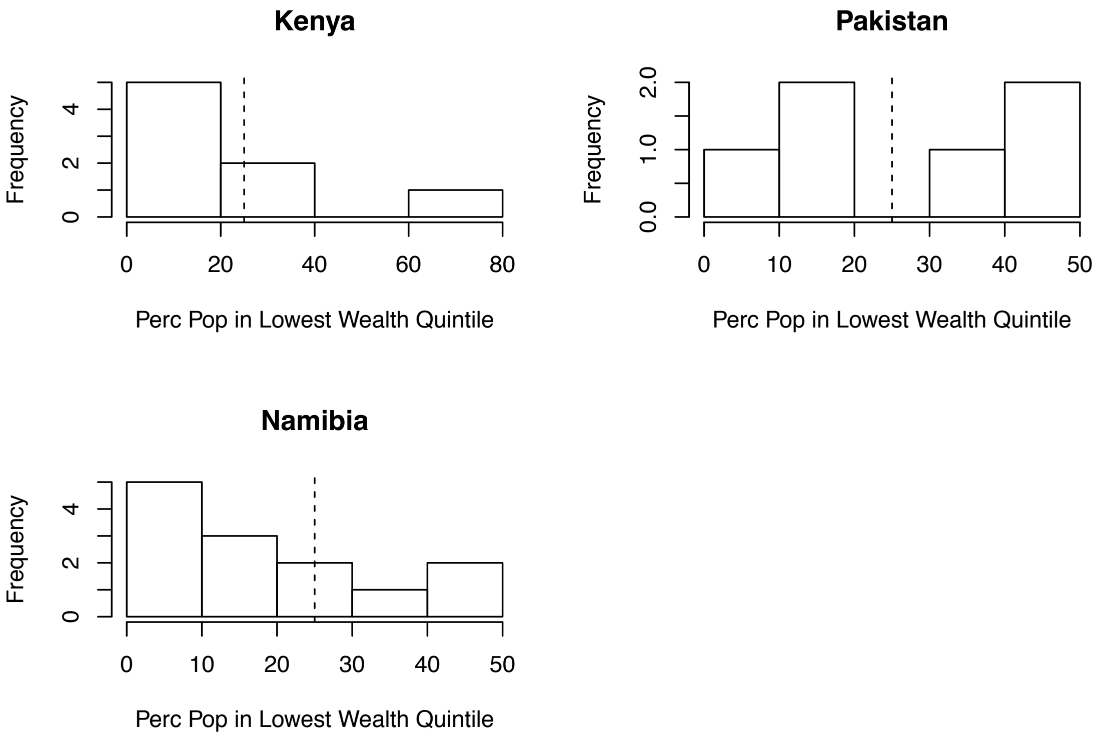

Supplementary Figure 11: The percentage of the population in the lowest wealth quintile per country.

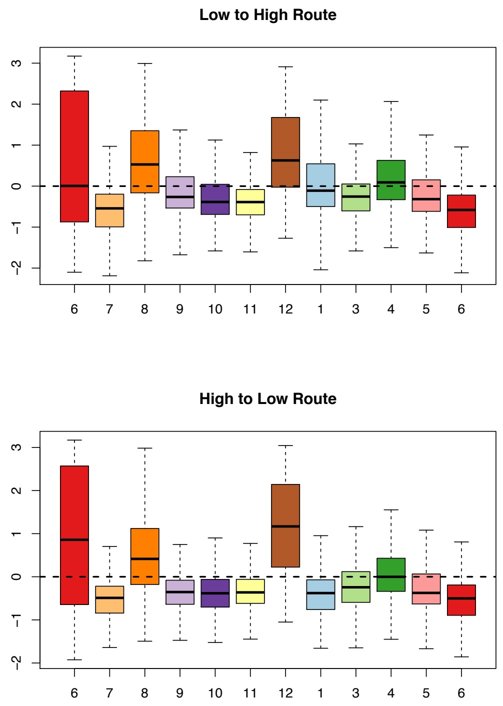

Supplementary Figure 12: The z-scores for different routes of travel between high and low income provinces per month in Kenya. For each route of travel between districts, the z-score of travel was calculated (see Materials and Methods). These routes were classified based on income levels of the origin and destination district. The distribution of monthly z-scores is shown.

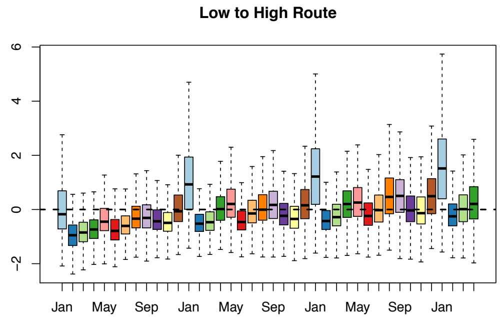

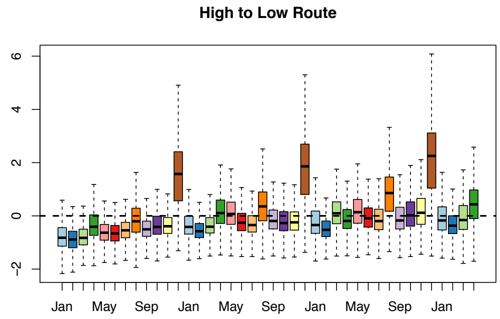

Supplementary Figure 13: The z-scores for different routes of travel between high and low income provinces per month in Namibia. For each route of travel between districts, the z-score of travel was calculated (see Materials and Methods). These routes were classified based on income levels of the origin and destination district. The distribution of monthly z-scores is shown.

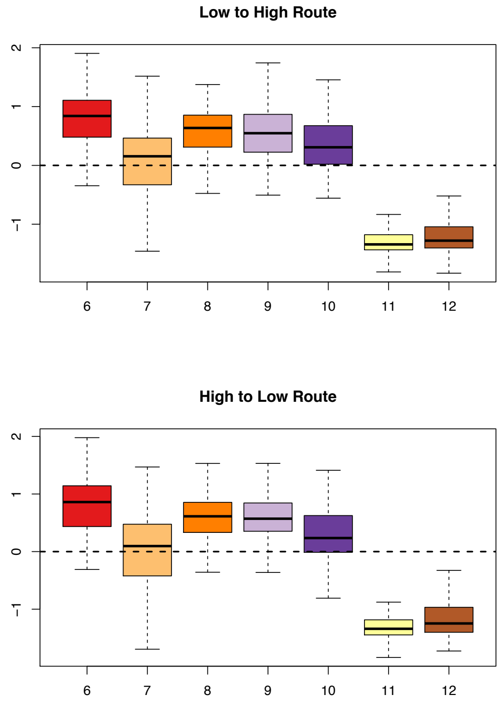

Supplementary Figure 14: The z-scores for different routes of travel between high and low income provinces per month in Pakistan. For each route of travel between districts, the z-score of travel was calculated (see Materials and Methods). These routes were classified based on income levels of the origin and destination district. The distribution of monthly z-scores is shown.

Supplementary Note 3: Simulating a general diffusion process

For each month, the total number of trips between all pairs of districts was calculated. These values formed a normalized (by the number of outgoing trips per location) travel matrix (N_{ij} - normalized trips between i and j) that was also converted into a quasi-distance matrix ($dist_{ij} = 1 - log\left(N_{ij}\right)$) between all pairs of districts per month [2]. Using these monthly distance matrices two connectivity measures were calculated. The weight connectance [3], equal to the weighted edge density divided by the total number of edges, was calculated.

Using these distance matrices, we also calculated the shortest paths between all pairs of districts to form a connectivity matrix. We performed a simple diffusion process on the monthly connectivity matrices. Given an identified starting location, in each time step a location is 'infected' if it is the closest (based on the shortest path) to all currently 'infected' locations. In this method, each location, once 'infected', remains infected for the remaining time steps. We ran each simulation until every district was infected. We started each simulation in the most populated district. We then compared the order districts become 'infected' between months. We calculated the z-score of the monthly order per location over the time period of the data.

Our main aim here was to illustrate that seasonal variation in the magnitude of travel as well as the direction of travel can substantially alter the spread of pathogens over the course of a year. In future applications, more detailed analyses encompassing the sequence of connectivity patterns over the course of a year, including the relative magnitude of travel in each season, and in the context of a particular pathogen biology, might allow prediction of the order of risk of pathogen spread, and could inform deployment of control efforts.

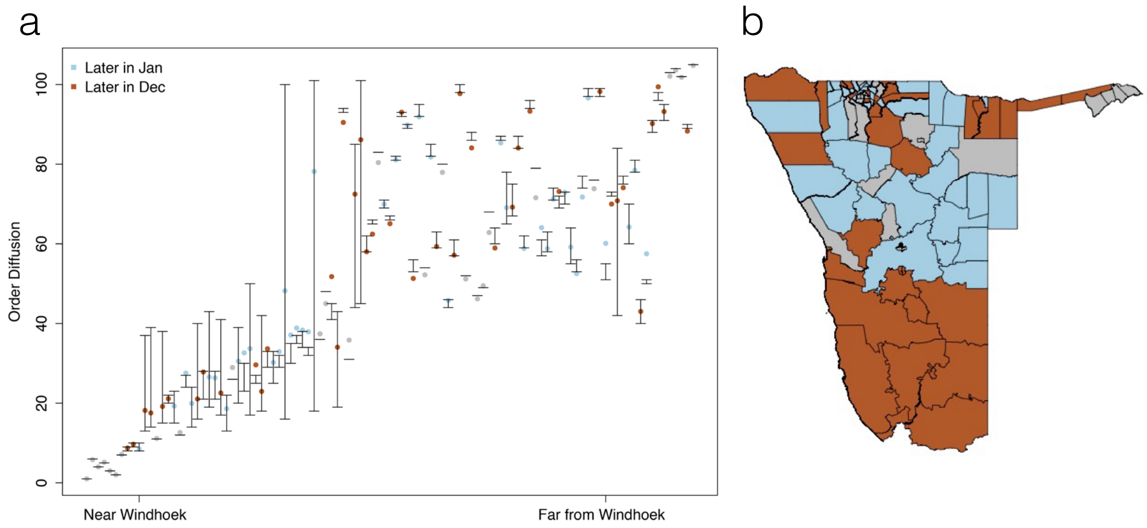

Supplementary Figure 15: The general results of a diffusion simulation. We performed a diffusion simulation starting in the most populated district (Windhoek East in Namibia). Based on the shortest path, a district becomes 'infected' in each time step if it is the closest (as measured by mobility) 'non-infected' district to all currently 'infected' districts. a) We compared the order that districts became 'infected' (y-axis, i.e., 1 means infected first, 20 means infected after 19 other districts, etc.) against physical distance (x-axis) from the starting infected location for each district in Namibia. The bars shows the range in ranks. If travel was purely determined by physical distance, every location would fall on a straight diagonal line. For places nearby Windhoek, the order they become 'infected' is roughly consistent with physical distance, i.e. those closest to Windhoek become 'infected' earlier. However, as distance from Windhoek increases, the order locations become 'infected' deviates from physical distance (does not fall on the straight diagonal line). The timing at which a district becomes infected will also depend on when during the year the pathogen was introduced, given seasonal fluctuations in mobility (we assume rapid spread, so retain the connectivity matrix for each month for the full course of the pathogens' spread). Given their striking differences, we focus on a comparison between January and December of 2013. On the plot (a), and the map (b), If a district became 'infected' faster following a January introduction of the pathogen, it is shown in blue, if it became 'infected' faster following a December introduction, it is shown in brown. Districts that had the same rank for introductions in either month are shown in grey. Locations in the southern part of the country were reached earlier following a January introduction of the pathogen. District boundaries were obtained from: www.diva-gis.org and were created using ArcGIS 10.3.

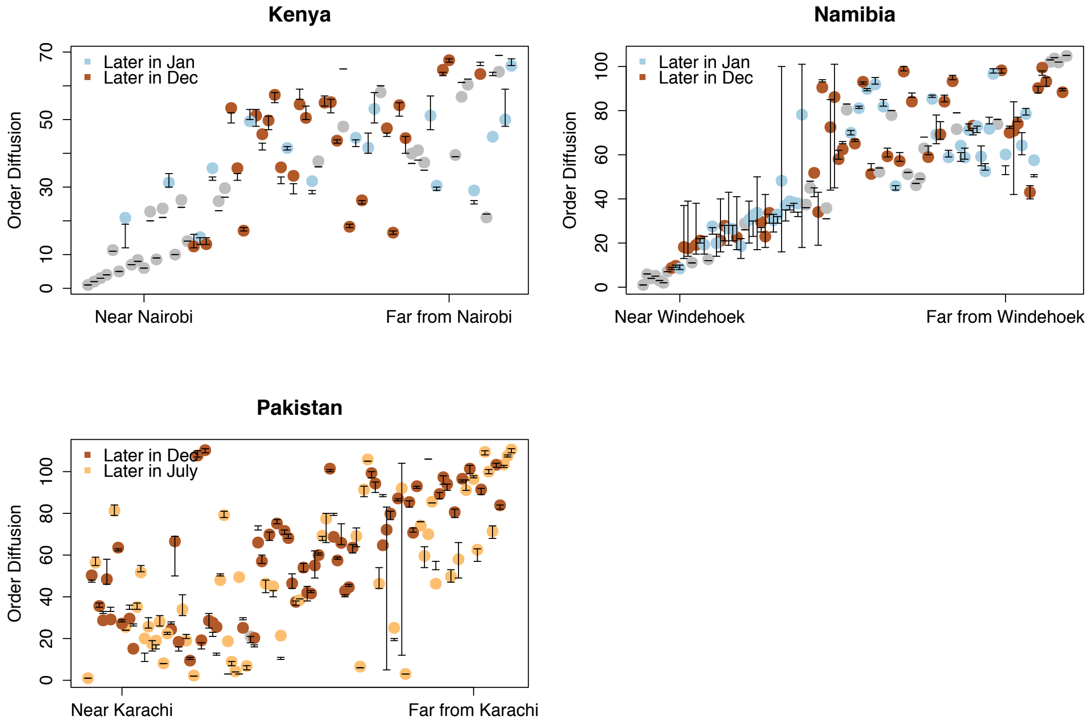

Supplementary Figure 16: The general results of a diffusion simulation for all three countries. We performed a simple diffusion simulation starting in the most populated district (Windhoek East in Namibia, Nairobi in Kenya, and Karachi in Pakistan). Using the method described in the Materials and Methods (and Fig. 4), we compared the order the districts became 'infected' (y-axis, i.e., 1 means infected first, 20 means infected after 19 other districts, etc.) against physical distance (x-axis) from the starting infected location for each district in each country. We then identified if the location became 'infected' later in December (brown) versus January (blue) or were the same order (grey), for Kenya and Namibia. Since the data set in Pakistan is over a shorter time frame in Pakistan, we compared the order a location became 'infected' in December versus July (yellow).

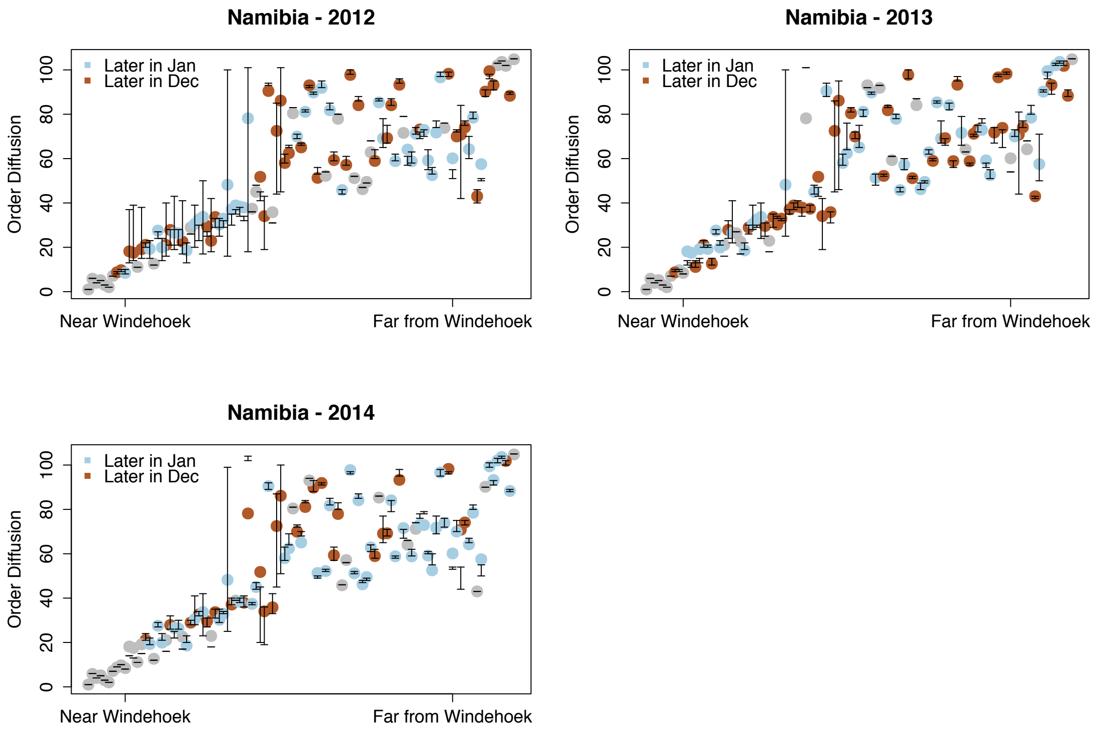

Supplementary Figure 17: The general results of a diffusion simulation for all three countries. We performed a simple diffusion simulation starting in the most populated district (Windhoek East in Namibia) for each year (2012 – 2014). Using the method described in the Materials and Methods (and Fig. 4), we compared the order the districts became 'infected' (y-axis, i.e., 1 means infected first, 20 means infected after 19 other districts, etc.) against physical distance (x-axis) from the starting infected location for each district in each country. We then identified if the location became 'infected' later in December (brown) versus January (blue) or were the same order (grey). In all three comparisons, results are similar to those shown in Supplementary Fig. 15.

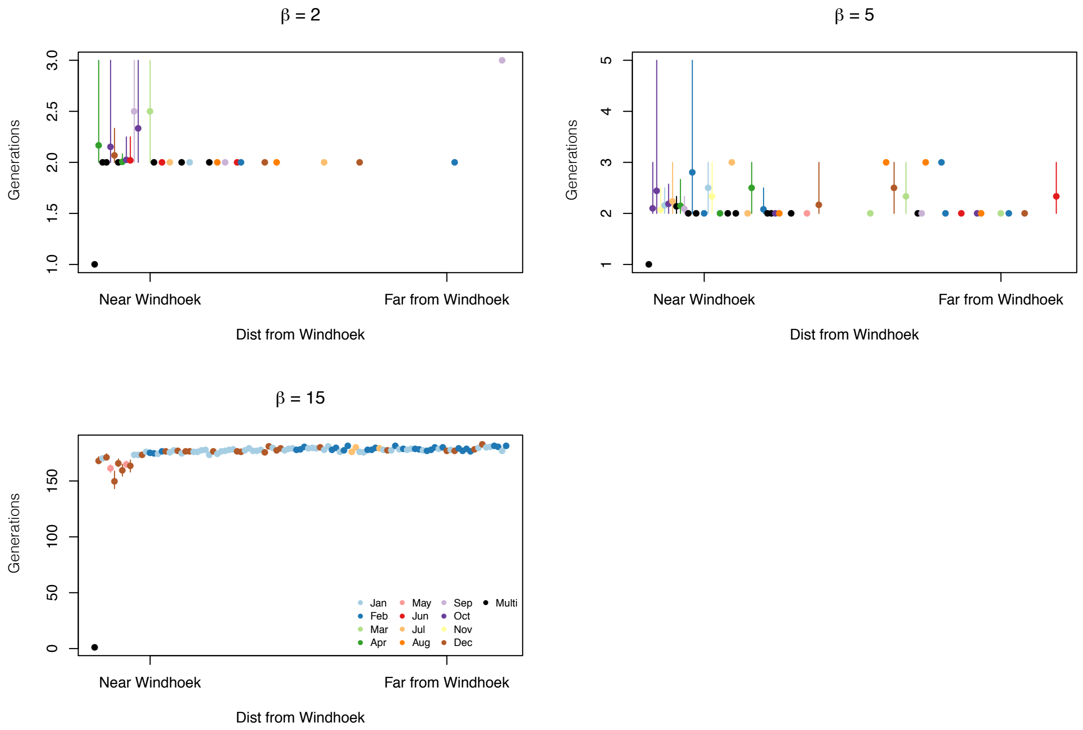

Supplementary Figure 18: Consequences of seasonal travel in a spatial diffusion model for a 5% proportion of the population susceptible. As in Fig. 4, we simulated spatial spread of a pathogen (see Materials and Methods) starting in the most populated district (Windhoek East in Namibia). Pathogen transmissibility, proportion of the population susceptible, and monthly connectivity value between districts defined a time varying hazard of introduction into each district. We compared the time (y-axis in days) each location became infected versus Euclidean distance from Windhoek (x-axis). Each district is colored by the slowest month (i.e. when the pathogen on average took the longest to reach a particular district). To explore the impact of different pathogen life histories, we compared varying magnitudes of transmission (β) ranging from low ($\beta = 2$) to intermediate ($\beta = 5$) to high ($\beta = 15$). The bars show the range in timings across the different months. We performed each hazard simulation 100 times per month and then average across simulations and months. For many locations, the pathogen will not reach the district, aside from those that become infected early on. For places nearby Windhoek when transmission is high, the time they become infected is consistent with physical distance, i.e. those closest to Windhoek become infected earlier. For locations nearby Windhoek, the pathogen reaches these locations the slowest in December when transmission is high, reflective of the decrease in travel from Windhoek East to nearby locations in this month. For majority of other districts have the latest relative timing in January or February since increases in these months to nearby districts decreases the amount of travel to all other districts.

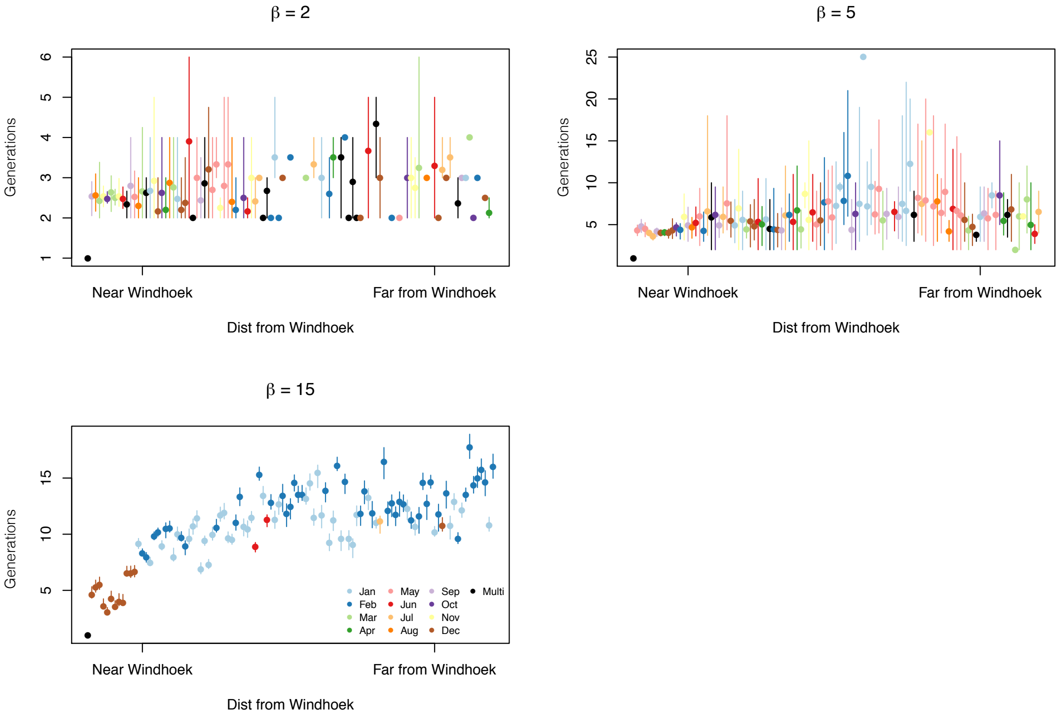

Supplementary Figure 19: Consequences of seasonal travel in a spatial diffusion model for a 20% proportion of the population susceptible. As in Fig. 4, we simulated spatial spread of a pathogen (see Materials and Methods) starting in the most populated district (Windhoek East in Namibia). Pathogen transmissibility, proportion of the population susceptible, and monthly connectivity value between districts defined a time varying hazard of introduction into each district. We compared the time (y-axis in days) each location became infected versus Euclidean distance from Windhoek (x-axis). Each district is colored by the slowest month (i.e. when the pathogen on average took the longest to reach a particular district). To explore the impact of different pathogen life histories, we compared varying magnitudes of transmission (β) ranging from low ($\beta = 2$) to intermediate ($\beta = 5$) to high ($\beta = 15$). The bars show the range in timings across the different months. We performed each hazard simulation 100 times per month and then average across simulations and months. For places nearby Windhoek when transmission is high, the time they become infected is consistent with physical distance, i.e. those closest to Windhoek become infected earlier. For locations nearby Windhoek, the pathogen reaches these locations the slowest in December when transmission is high, reflective of the decrease in travel from Windhoek East to nearby locations in this month. For majority of other districts have the latest relative timing in January or February since increases in these months to nearby districts decreases the amount of travel to all other districts.

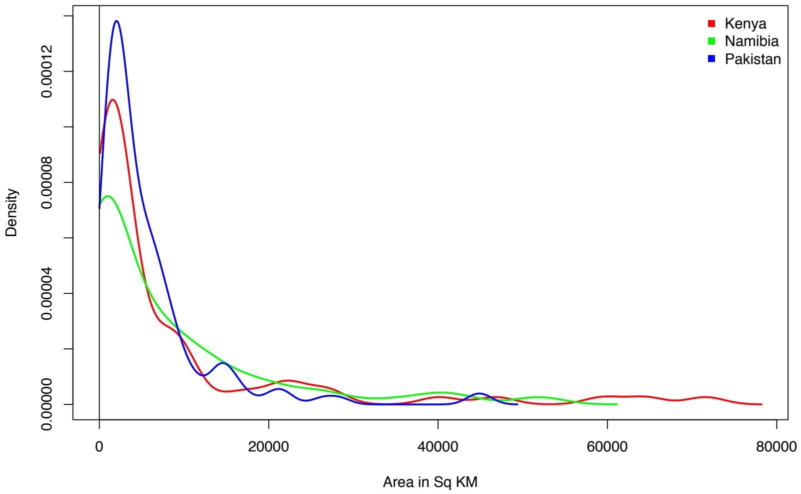

Supplementary Figure 20: The distribution of district areas in each country. In Kenya and Namibia, the area of districts are similar (mean value in Kenya: 11,500 km^2, Namibia: 8,000 km^2) with similar maximum areas (Kenya: 72,000 km^2, Namibia: 52,500 km^2). The areas of districts in Pakistan are on average smaller (mean: 5,700 km^2, max: 45,000 km^2).

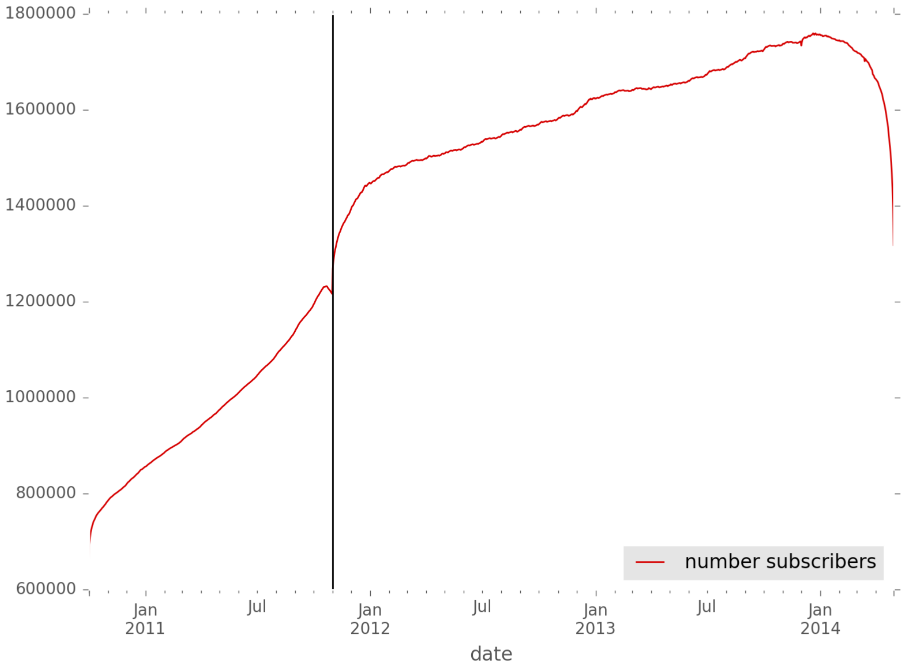

Supplementary Figure 21: The number of subscribers in Namibia over the course of the data set analyzed. The number of subscribers in Namibia substantially changes over the course of the data set from fewer than 1 million in early 2011 to nearly 2 million in early 2014. As a result of these large subscriber changes, we normalized each yearly data set by the total number of subscribers in that year.

Supplementary References

1. USAID. Available at: statcompiler.com. Accessed 08/15/2017.
2. Brockmann D, Helbing D. The hidden geometry of complex, network-driven contagion phenomena. Science. 2013;342(6164):1337-42.
3. Tylianakis JM, Tscharntke T, Lewis OT. Habitat modification alters the structure of tropical host-parasitoid food webs. Nature. 2007;445(7124):202-5
